# Successful rivaroxaban and doxycycline therapy for *Mycoplasma pneumonia* with pulmonary embolism in children: a case report

**DOI:** 10.3389/fped.2025.1449493

**Published:** 2025-02-12

**Authors:** Fang Xiaoqian, Lu Hemin

**Affiliations:** Department of Pediatrics Department, Dongyang People’s Hospital, Dongyang, Zhejiang, China

**Keywords:** *mycoplasma*, pulmonary embolism, rivaroxaban, doxycycline, case report

## Abstract

The mortality of pulmonary embolism in children is high, and there is no standardized treatment protocol. We present a case of successful treatment of *Mycoplasma pneumoniae* (*M pneumoniae*) with pulmonary embolism in a child using rivaroxaban and doxycycline, thereby exploring a more appropriate treatment option. A 10-year-old male presented with fever, cough, and chest pain as the main symptoms. *M pneumoniae* polymerase chain reaction of bronchoalveolar lavage fluid was positive, and computed tomography angiography indicated pulmonary embolism. Azithromycin, doxycycline, and piperacillin-tazobactam were administered sequentially for infection control, while methylprednisolone was given to control inflammation and heparin and rivaroxaban for sequential treatment, resulting in a satisfactory recovery.

## Introduction

The incidence rate of pulmonary embolism in children has been increasing in recent years. The incidence rate of pulmonary embolism in children in the community ranges from approximately 1.4 to 9 cases per one million (1), and in American children, it increased by 200% between 2001 and 2014 (2). The mortality of pulmonary embolism is high in children, with reports indicating up to 26% (3). Currently, there is no standardized treatment for pulmonary embolism caused by *Mycoplasma pneumoniae* (*M pneumoniae*) in children. This study presents a case of *M pneumoniae* accompanied by pulmonary embolism in a pediatric patient who was successfully treated with rivaroxaban and doxycycline. Promptly initiating treatment in children with this dual diagnosis resulted in favorable outcomes, such as shortened hospitalization, improved safety, and good patient adherence.

## Case description

**Figure 1:**
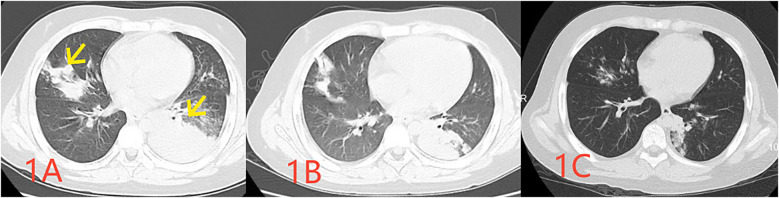
Pulmonary CT images obtained at 1 week (**A** indicating the infected lesion with a yellow arrow), 2 weeks (**B** indicating absorption at the site of infection), and 1 month (**C** demonstrating the essentially absorbed focus of infection) following the disease onset.

A previously healthy 10-year-old male child was admitted to our hospital with a history of fever and cough for 8 days and chest pain for 1 day. Initial examination revealed a C-reactive protein (CRP) level of 112 mg/L (reference range: 0–10 mg/L), and pulmonary computed tomography (CT) revealed multiple inflammatory areas in both lungs (Figure 1A).

Before admission, the patient received ceftriaxone for 1 day, azithromycin for 5 days, and concurrently, methylprednisolone for 4 days without any improvement.

One Day before admission, the patient developed chest pain, and reexamination revealed a CRP level of 133 mg/L. A pharyngeal swab test indicated a positive *M pneumoniae* polymerase chain reaction (PCR). A repeat pulmonary CT demonstrated multiple inflammatory areas in both lungs, accompanied by focal consolidation (Figure 1B). During the course of the disease, the child exhibited no other symptoms. At the time of examination, the child weighed 50.5 kg, measured 142 cm in height, had a respiratory rate of 40 breaths per minute, coarse breath sounds, and audible wet rales in both lungs.

No other pathogenic bacteria were detected following admission. Two days post-admission, a bronchoscopy at our hospital found thick sputum in the right bronchus. Bronchoalveolar lavage fluid tested positive for pneumonia type *M pneumoniae* via PCR, with no other pathogens like tuberculosis detected.

**Figure 2:**
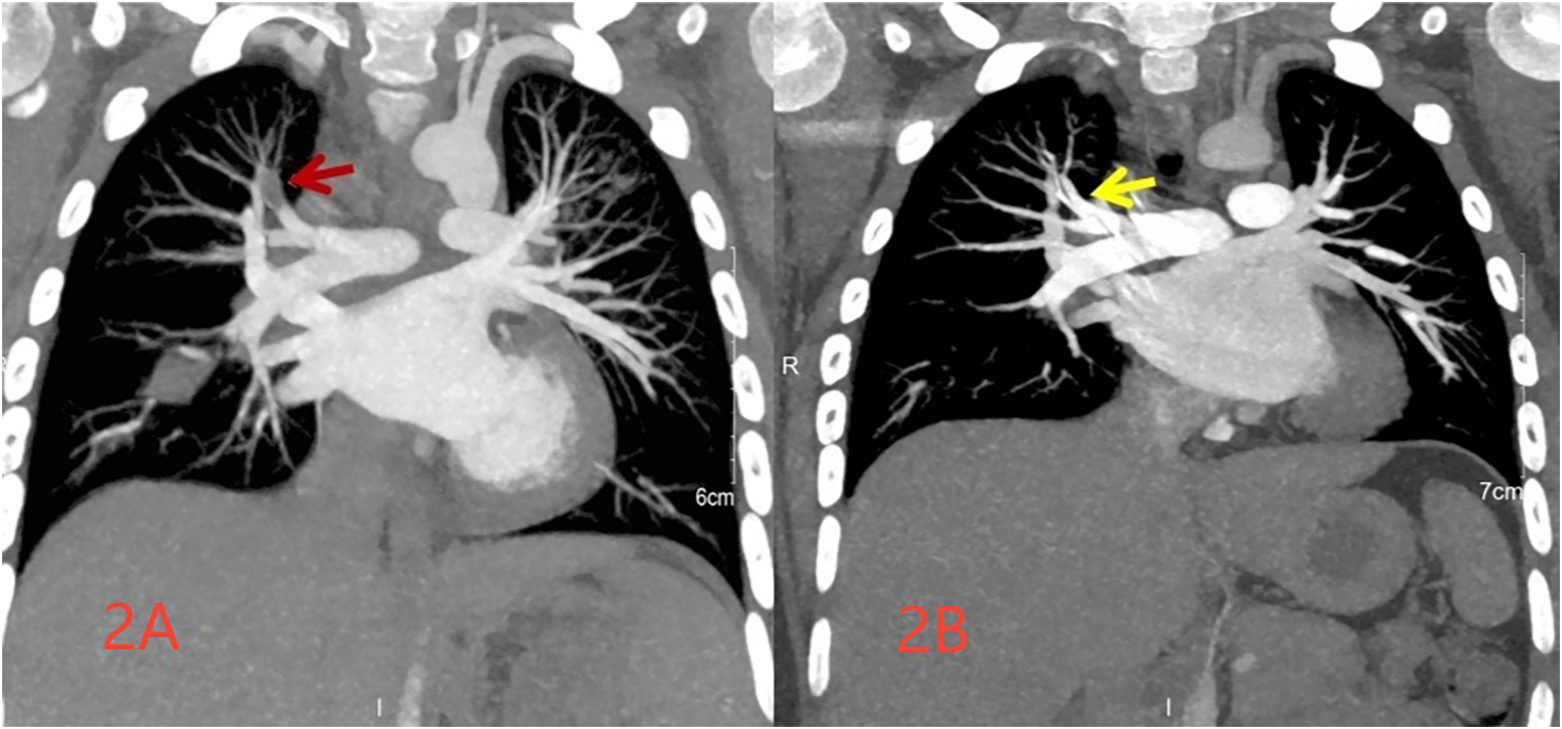
Pulmonary CTA images obtained at 10 days (**A** highlighting embolus formation in the lung with a red arrow) and 3 months (**B** indicating absorption of the pulmonary embolus post-treatment with a yellow arrow) following the disease onset.

Following admission, the patient received azithromycin (10 mg/kg/day for 2 days), followed by doxycycline (2 mg/kg/dose every 12 h for 10 days) combined with piperacillin-tazobactam (4.5 g/dose every 8 h for 9 days) for infection management, and methylprednisolone for anti-inflammatory treatment. On the second day of admission, the patient's chest pain disappeared, and body temperature normalized. D-dimer levels were measured at 16.88 μg/ml (reference range: <0.5 μg/ml), and enoxaparin calcium was administered subcutaneously at 4,100 International Units per day. On the third day, pulmonary artery computed tomography angiography (CTA) revealed a right upper pulmonary artery branch embolism (Figure 2A). Heparin was discontinued, and oral rivaroxaban (10 mg/time orally twice daily for one month, followed by 15 mg/time once daily for 2 months) was administered. There were no other symptoms, such as swelling or pain in the limbs, observed throughout the process. The following laboratory parameters were monitored: Hemoglobin (106–136 g/L; reference range: ≥120 g/L), Platelet count (173–352 × 109/L; reference range: 100–453 × 109/L), International Normalized Ratio (INR; 1.1–1.4; reference range: 0.8–1.3), thrombin time (TT) (13.6–16.3 s; reference range: 13.6–16.3 s), plasma prothrombin time (PT) (13.6–6.3 s; reference range: 13.6–6.3 s), activated partial thromboplastin time (APTT) (39–50 s; reference range: 28–44 s), alanine aminotransferase (49 U/L; reference range: 9–50 U/L), aspartate aminotransferase (48 U/L; reference range: 9–50 U/L), urea (3.57 mmol/L; reference range 3.1–8 mmol/L), and creatinine (49 µmol/L; reference range: 57–97 µmol/L).

However, two weeks after discharge, the patient experienced an episode of increased coughing, with a CRP level of 63.22 mg/L. The D-dimer level was measured at 0.54 μg/L, and a follow-up pulmonary CT indicated resolution of the previous bilateral multiple inflammation, with new cavities in the lesion of the left lower lobe (Figure 1C). After 10 days of additional treatment with oral cefixime, the cough resolved with no recurrence. Three months after discharge, the patient's D-dimer level was 0.22 μg/ml, and no cough symptoms were present. The original embolic lesions were absorbed, and pulmonary CTA revealed no pulmonary embolism (Figure 2B). The child was monitored for 6 months after discontinuing rivaroxaban without any discomfort (Table 1).

**Table 1:** Disease changes and diagnosis and treatment process.

	Clinical manifestation	CRP (mg/L)	D-dimer (*μ*g/ml)	Other tests	Antibiotic	Anticoagulant	Methylprednisolone
Day1–2	Fever&cough	112			Ceftriaxone		
Day 3–6				IgM (+)	Azithromycin		40 mg/dose ivgtt every day
Day 7	Chest pain	133		Mycoplasma PCR (+) CT (Figure 1A)			
Day 8 (be hospitalized)	Wet rales	108	16.9		Azithromycin and piperacillin-tazobactam	Enoxaparin calcium	40 mg/dose ivgtt every 12 h
Day 9	No chest pain, no fever			Bronchoscopy: mycoplasma PCR (+)			
Day 10		41	2.93		Doxycycline and piperacillin-tazobactam		
Day 11	No rales			CTA (Figure 2A)			
Day 12			2.69			Rivaroxaban for 3 months	40 mg/dose ivgtt every day
Day 13–16		13	1.76	CT (Figure 1B)			
Day17 (hospital discharge)	Occasional cough	14			Doxycycline (Day 10–20)		20 mg/dose orally twice daily (Day 16–22)
2 weeks after discharge	Cough increases again	63	0.54	CT (Figure 1C)	Cefixime for 10 days		
1 month after discharge	No cough	3.4	0.22				
3 months after discharge	Physical health		0.22	CTA (Figure 2B)		Drug withdrawal	

## Discussion

Children have a significantly increased incidence of pulmonary embolism and a high mortality rate (1–3). *M pneumoniae* is one of the most common pathogens in children (4) and can directly or indirectly cause pulmonary embolism (5). Reports of pulmonary embolism caused by *M pneumoniae* in children have significantly increased in recent years (6, 7).

The exact pathogenesis of *M pneumoniae* is unclear. It may involve cytokine induction causing vascular damage and obstruction, or lipoprotein sugars from *M pneumoniae* inducing procoagulant activity, thereby resulting in a hypercoagulable state (5, 8). When coagulation occurs, thrombin activates the fibrinolytic system, resulting in the formation of D-dimer (8). Previous studies have identified D-dimer as an independent risk factor for *M pneumoniae* associated with pulmonary embolism (9, 10). In our case, we examined a child with pulmonary embolism and found no symptoms of embolism. We monitored coagulation markers (INR, APTT, PT, TT, and D-dimer) and observed a significant increase in D-dimer levels during the embolism, which normalized once the embolism resolved. However, additional examinations were not conducted to further investigate the underlying cause of pulmonary embolism formation, and no specific coagulation defects have been identified.

Anticoagulation is crucial for managing children with *M pneumoniae* and embolism; however, no standardized treatment protocol exists for complicated cases of pulmonary embolism. Currently, the primary drugs used for treating pulmonary embolism in children include heparin, vitamin K antagonists such as warfarin, and direct oral anticoagulants (DOACs), including rivaroxaban, apixaban, and dabigatran (6, 7, 11, 12).

Although the conventional anticoagulant warfarin is widely utilized for the prevention and treatment of deep vein thrombosis, it requires regular monitoring of the INR and is affected by dietary factors, such as the consumption of spinach and wolfberry, which can elevate the risk of bleeding (13). Furthermore, the efficacy of warfarin in treating pediatric patients with *M pneumoniae* complicated by pulmonary embolism remains inconsistent. One child in the United States (14), four in Shanghai (6), and thirty-eight in Beijing (15) exhibited a good prognosis with *M pneumoniae* and pulmonary embolism; however, a fatal case was reported in Zhengzhou (16). However, among seven cases of *M pneumoniae* complicated by pulmonary embolism in Jilin, China, five children revealed no response to heparin and warfarin treatment and underwent surgical treatment, whereas two patients died of acute respiratory distress syndrome after surgery (17).

The DOAC rivaroxaban has been approved for use in children in the United States (18). Studies have indicated that DOACs have sufficient safety and efficacy in adolescents (11). In a Phase III trial investigating the safety and efficacy of rivaroxaban for treating venous thrombosis in children, the results were similar to those observed in adults (19). A real-world study demonstrated that rivaroxaban was safe for treating venous thrombosis in children, with a thrombosis recurrence rate of 3.6% (20). Studies have revealed that adult patients taking rivaroxaban for deep vein thrombosis or pulmonary embolism experience shorter hospital stays, lower costs, and a lower rate of post-thrombotic syndrome (0.53% vs. 0.55%) compared to those taking warfarin (21, 22). However, reports on rivaroxaban for treating *M pneumoniae* with pulmonary embolism in children have focused on Tianjin, China (7, 23, 24). A study demonstrated nine cases of children diagnosed with *M pneumoniae* and pulmonary embolism at Tianjin Children's Hospital between 2,018 and 2021 (7). These children received a sequential treatment regimen involving low-molecular-weight heparin and rivaroxaban. The follow-up period ranged from 0.5–9 months, during which no cases of thrombosis were observed (7).

The patient was discharged after 9 days of hospitalization. During the three months of treatment, the child was monitored only for coagulation and D-dimer levels several times while receiving rivaroxaban. After 6 months of rivaroxaban treatment, the child was free of cough and other symptoms. A CTA review indicated the disappearance of the embolism, and both the D-dimer levels and coagulation function were normal, enabling the child to engage in appropriate physical activity. The child's hospitalization lasted 9 days, significantly shorter than the typical duration for those on warfarin [Chen et al., 13–23 days (6); Song et al., at least 15–21 day s (7)], and there was no need for regular INR monitoring. Family members expressed high satisfaction with the entire treatment process and reported a good prognosis.

## Conclusion

Rivaroxaban is a safe and convenient treatment option for pulmonary embolism resulting from *M pneumoniae* in children, with a positive prognosis.

## Data Availability

The original contributions presented in the study are included in the article/Supplementary Material, further inquiries can be directed to the corresponding author.
